# Immobility in the sedentary plant-parasitic nematode *H*. *glycines* is associated with remodeling of neuromuscular tissue

**DOI:** 10.1371/journal.ppat.1007198

**Published:** 2018-08-16

**Authors:** Ziduan Han, Sita Thapa, Ursula Reuter-Carlson, Hannah Reed, Michael Gates, Kris N. Lambert, Nathan E. Schroeder

**Affiliations:** 1 Department of Crop Sciences, University of Illinois at Urbana-Champaign, Urbana, IL, United States of America; 2 Neuroscience Program, University of Illinois at Urbana-Champaign, Urbana, IL, United States of America; University of California Riverside, UNITED STATES

## Abstract

The sedentary plant-parasitic nematodes are considered among the most economically damaging pathogens of plants. Following infection and the establishment of a feeding site, sedentary nematodes become immobile. Loss of mobility is reversed in adult males while females never regain mobility. The structural basis for this change in mobility is unknown. We used a combination of light and transmission electron microscopy to demonstrate cell-specific muscle atrophy and sex-specific renewal of neuromuscular tissue in the sedentary nematode *Heterodera glycines*. We found that both females and males undergo body wall muscle atrophy and loss of attachment to the underlying cuticle during immobile developmental stages. Male *H*. *glycines* undergo somatic muscle renewal prior to molting into a mobile adult. In addition, we found developmental changes to the organization and number of motor neurons in the ventral nerve cord correlated with changes in mobility. To further examine neuronal changes associated with immobility, we used a combination of immunohistochemistry and molecular biology to characterize the GABAergic nervous system of *H*. *glycines* during mobile and immobile stages. We cloned and confirmed the function of the putative *H*. *glycines* GABA synthesis-encoding gene *hg-unc-25* using heterologous rescue in *C*. *elegans*. We found a reduction in gene expression of *hg-unc-25* as well as a reduction in the number of GABA-immunoreactive neurons during immobile developmental stages. Finally, we found evidence of similar muscle atrophy in the phylogenetically diverged plant-parasitic nematode *Meloidogyne incognita*. Together, our data demonstrate remodeling of neuromuscular structure and function during sedentary plant-parasitic nematode development.

## Author summary

The sedentary plant-parasitic nematodes are an economically devastating group of pathogens. They are capable of modifying their host’s metabolism by establishing a permanent feeding site within the plant roots. Following infection, sedentary nematodes remain at their feeding site where they are only capable of slight movements associated with feeding. Upon reaching adulthood, females continue to remain immobile at their feeding site, while adult males regain mobility to search for females. Using both microscopic observation and examination of gene function, we found that immobility is associated with changes to the muscle and neurons controlling movement. Interestingly, we found that males are capable of reversing these changes upon reaching adulthood. Our findings may be used to develop targeted control strategies for these devastating parasitic nematodes while avoiding potential off-target effects to beneficial nematodes that do not show this unusual mode of development and behavior.

## Introduction

**Fig 1:**
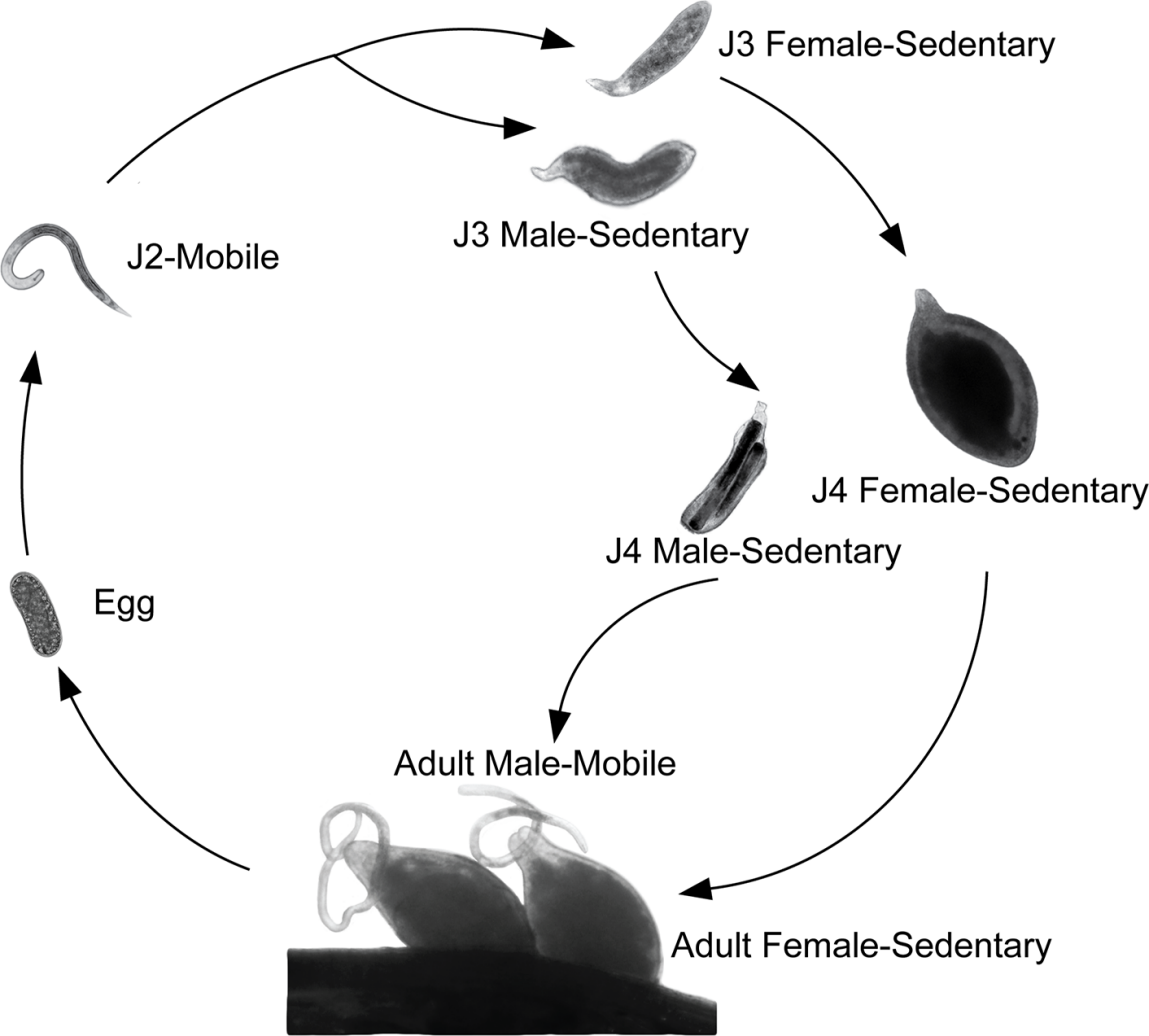
The life cycle of *H*. *glycines*. *H*. *glycines* hatch as mobile second-stage juveniles (J2s), migrate to the host roots, infect and initiate feeding. Following the initiation of feeding, the body diameter increases and the nematodes become sedentary. J3s remain sedentary and continue feeding. During late J3, we are able to distinguish sexes. Following the molt to J4, males begin remodeling into a vermiform shape while females continue to feed and grow in width. Following the final molt, adult males are fully mobile and seek out females to inseminate. Adult females remain immobile and continue to feed. Each life stage is not represented to scale.

The sedentary plant-parasitic nematodes are among the most damaging pathogens to agricultural crop production [1]. For example, the soybean cyst nematode, *Heterodera glycines*, accounts for over a billion dollars in yield loss yearly [2]. *H*. *glycines* hatches as an infective, mobile second-stage juvenile (J2) (Fig 1). Following infection, *H*. *glycines* establishes a multinucleated feeding site and develops from a vermiform J2 to lemon-shaped and sausage-shaped J4 females and males, respectively (Fig 1). While feeding, both sexes are restricted in movement to slight head motion at their feeding site [3]. During J4, males undergo extensive morphological remodeling back to a vermiform morphology. Following the final molt, the adult male is fully mobile, while the female remains lemon-shaped and immobile.

The structural basis for the loss of mobility and male-specific resumption of mobility in sedentary nematodes is not clear. Several authors have speculated that immobility is due to muscle degeneration [4–6]. For example, Bird [6] demonstrated the immobility of the sedentary nematode *Meloidogyne javanica* through careful dissection of nematodes from roots and subsequent behavioral assays. He later speculated that loss of mobility was due to somatic muscle atrophy [7]. An early histological examination of *Meloidogyne hapla* noted an absence of most somatic muscle fibers in the adult female and suggested that loss of muscle was a result of sedentary behavior [4]. However, this description was limited by the resolution of light microscopy and did not examine changes during development. Nor did this work account for possible changes in the nervous system that could impact mobility.

The structure of body wall muscles during mobile stages of sedentary plant-parasitic nematodes are similar to *Caenorhabditis elegans* ([8–10] and herein). The body wall muscle comprises four quadrants with striated muscle fibers oriented in an oblique angle to the longitudinal axis. In *C*. *elegans*, the majority of body wall muscle is innervated by a series of motor neurons in the ventral nerve cord (VNC) (S1 Fig) [11]. We recently demonstrated substantial variability in the number of VNC neurons among nematodes, including differences between J2 *H*. *glycines* and *C*. *elegans* [12]; however, the specific wiring pattern of somatic muscle in sedentary plant-parasitic nematodes is unknown. In *C*. *elegans*, movement is generated through muscle contractile force propagated to the outer cuticle through a thin basal lamina and epidermal layer via fibrous organelles [13]. Muscle contractions are produced via the innervation of excitatory cholinergic and inhibitory GABAergic VNC motor neurons [14,15].

Within the phylogenetic clade comprising most plant-parasitic nematodes, the sedentary life-style has arisen at least twice [16,17]. While having a similar life-cycle, the root-knot (Meloidogynidae) and cyst (Heteroderidae) nematodes are phylogenetically diverged. The sedentary nematodes are more fecund and considered more damaging than their phylogenetically closest “migratory” plant-parasitic nematode relatives [1,18]. An understanding of the neuromuscular modifications that occur during sedentary nematode development could provide novel targets for control. For example, the resumption of mobility by male cyst nematodes could be targeted without affecting non-target nematodes that do not undergo this unusual development.

Here, we conduct a detailed developmental examination of the structural basis for changes of mobility in the sedentary species *H*. *glycines*. Using light and electron microscopy, we describe the anatomical changes to the neuromusculature and surrounding epidermis of *H*. *glycines* during the transition to immobility and the resumption of mobility in males. We characterize the GABAergic system during *H*. *glycines* post-hatch development. Finally, we examine whether similar changes to body wall muscles occur in the phylogenetically diverged root-knot nematode *Meloidogyne incognita*. Our results demonstrate extensive remodeling of neuromuscular tissue in sedentary plant-parasitic nematodes.

## Results

### *H*. *glycines* body wall muscles degenerate and undergo sex-specific renewal during development

**Fig 2:**
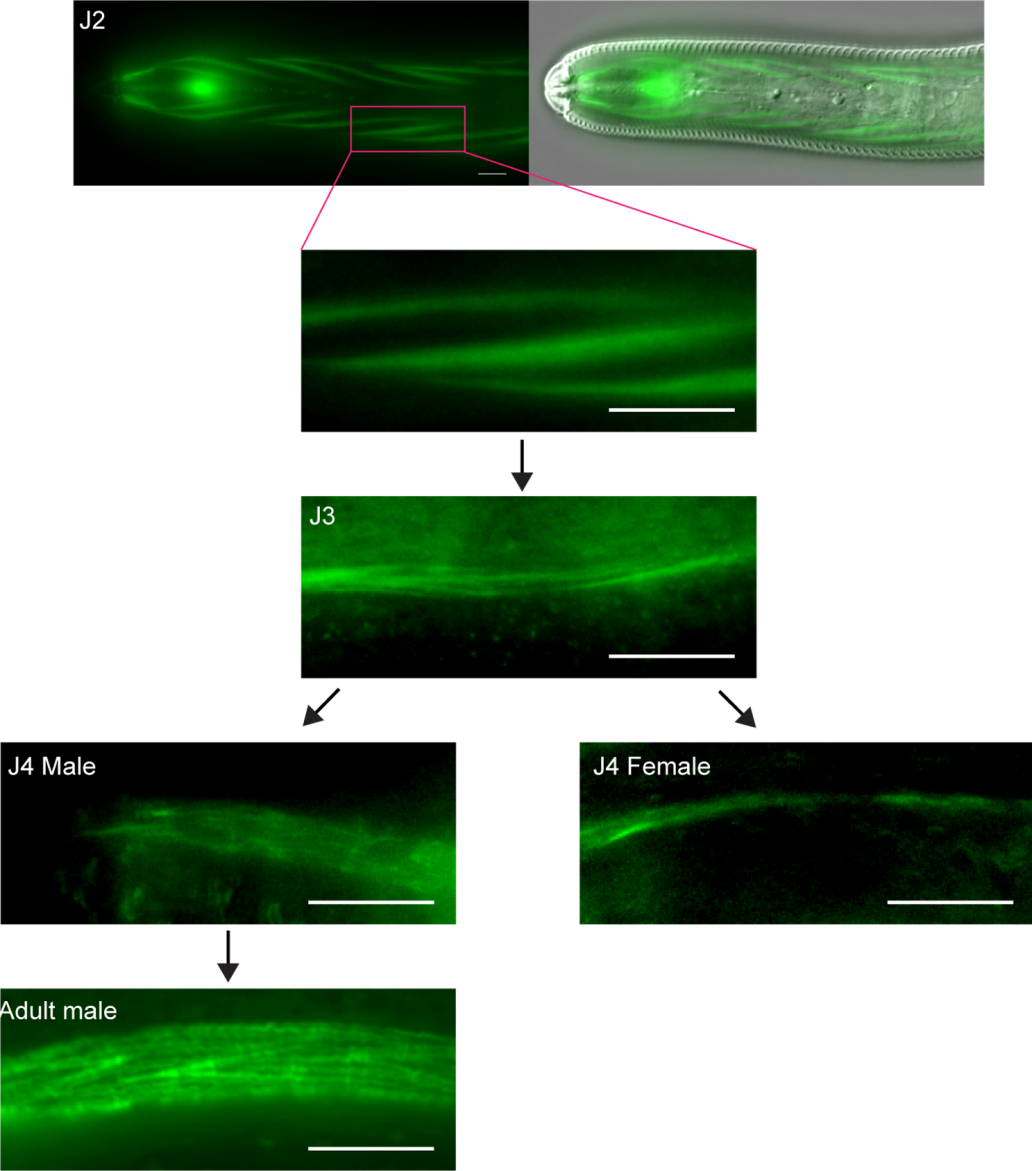
Body wall muscles atrophy during sedentary stages. Fluorescent micrographs of phalloidin-stained *H*. *glycines*. The J2 body wall muscles (image from head region) contain actin-enriched striated filaments obliquely oriented to the longitudinal axis. In J3, the diameter of muscle filaments is smaller than in J2 and lacks a well-defined organization. The phalloidin-stained body wall muscle filaments are thicker in J4 males compared with J3. In the adult male, the body wall muscles contain additional filaments, but are otherwise similar to J2 body wall muscles. In J4 females, the size of the actin-enriched muscle filaments is further reduced compared to J3s. Also see S1 Fig. Scale bars, 5 μm.

**Fig 3:**
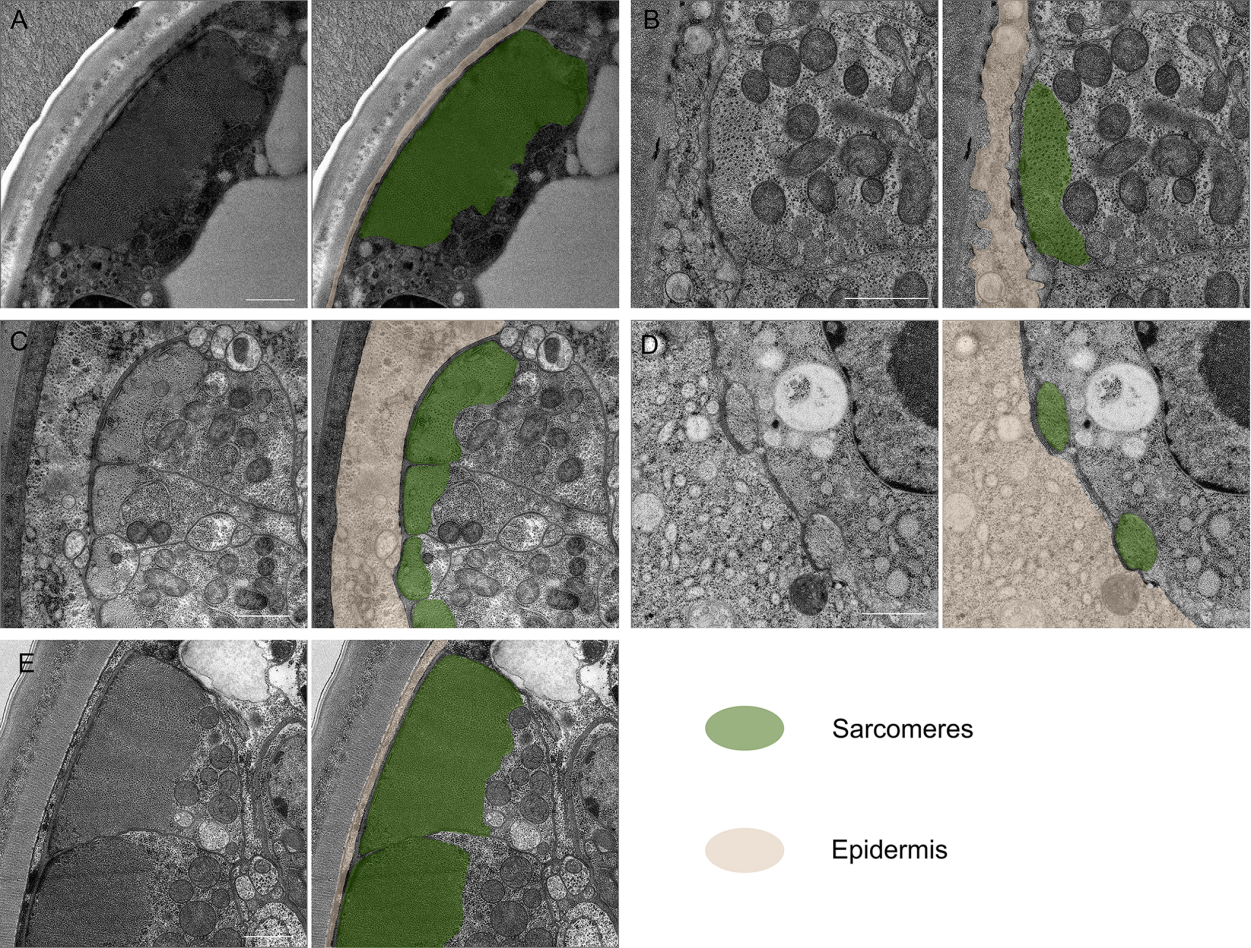
*H*. *glycines* body wall muscle undergoes ultrastructural changes during development. Transverse TEM sections of body wall muscle in the midbody of *H*. *glycines* (left) and with pseudocolor overlay (right). (A) Mobile J2 *H*. *glycines* have intact muscle separated from the cuticle by a thin layer of epidermis. (B) Sedentary J3 muscle shows signs of atrophy including fewer thick and thin filaments and a thickening of epidermis. (C) J4 male muscle comprise distinct sarcomeres, but with reduced numbers of thick filaments and an epidermal thickness similar to J3s. (D) J4 females undergo further atrophy of sarcomeres and increased thickening of the epidermis beyond that seen in J3s. (E) The muscles of adult males are similar in orientation and size to J2 muscle. Scale bar, 1 μm.

To test whether the immobility of sedentary nematodes was due to degeneration of body wall muscles [4,5,7], we first analyzed the somatic muscle tissue in the mobile J2 stage of *H*. *glycines* using both the F-actin binding fluorescent probe phalloidin as well as transmission electron microscopy (TEM). Mobile J2 *H*. *glycines* somatic muscle comprise rhomboid-shaped cells longitudinally arranged in four quadrants along the length of the body (Fig 2 and S1 Fig). Each quadrant contains two rows of overlapping muscle cells and each cell contains striations obliquely oriented to the longitudinal axis. Using TEM on transverse sections following high-pressure freezing and freeze substitution, we found that individual muscle cells in J2 *H*. *glycines* comprise multiple sarcomeres separated from the cuticle by a thin basal lamina and epidermis (Fig 3A). As previously shown [8,19–21], J2 sarcomeres are arranged into obvious A- and I-bands with thick and thin filaments. The non-contractile region of body wall muscles is enriched with mitochondria. Overall, our light and electron microscopy images correspond to previous data from mobile stages of *H*. *glycines* and indicate a similar muscle structure to *C*. *elegans*.

Within six days following infection, *H*. *glycines* molts to a J3 [22]. We found that phalloidin-stained J3 *H*. *glycines* somatic muscles are disorganized and smaller than in mobile J2s (Fig 2). The body wall muscles lose their rhomboid shape and the pattern of two-row cells in each quadrant is no longer recognizable. Our TEM examination of immobile J3s also demonstrated a shrinkage of muscle and sarcomere disorganization (Fig 3B and Table 1). The sarcomeres of immobile J3s lack distinguishable A- and I-bands. Furthermore, the number of thick and thin filaments appears reduced in J3s compared with J2s. Sexual differentiation of *H*. *glycines* is first visible in late-J3 (personal observation and [23]); however, we did not observe any obvious sex-based differences in muscle structure between male and female J3s suggesting that both sexes undergo muscle atrophy. In addition to structural changes to the muscle itself, we observed that the body wall muscles are displaced internally away from the cuticle in J3 *H*. *glycines*. This displacement is correlated with a corresponding thickening of the epidermis (Fig 3B and Table 1). In *C*. *elegans*, the force of body wall muscle contraction is transduced through a thin epidermal layer via attachment to the outer cuticle [13,24]. Loss of mobility in *H*. *glycines* may be due to a combination of muscle atrophy as well as an inability to transduce contractile force to the outer cuticle. We found that the body wall head muscles and esophageal muscles of immobile stages are intact (S2A Fig). This observation is consistent with the previously reported feeding behavior of cyst nematodes and suggests that muscle atrophy is cell-specific and not due to a generalized atrophy as seen in sarcopenia of *C*. *elegans* [25,26].

**Table 1:** Ultrastructural measurements of *H*. *glycines*.

	Sarcomere percent area^1^	Epidermal width (μm)
J2	4.75±1.73	0.143±0.049
J3/J4	0.13±0.05	2.1±2.5
J4 male	1.55±1.32	0.36±0.014
Adult male	10.4±2.3	0.115±0.007

^1^ Average percent sarcomere area ± standard deviation was calculated by measuring the sarcomere area in transverse TEM sections divided by the total cross section body area x 100. Sections from at least two animals were examined.

J4 *H*. *glycines* females continue to feed and grow in width, while J4 males begin remodeling back into a vermiform shape (Fig 1). In phalloidin-stained J4 females (Fig 2), we detected faint stripes underlying the epidermis suggestive of muscle filaments. Interestingly, nuclear staining revealed a row of nuclei, similar in size and shape to muscle nuclei, immediately underlying the phalloidin stripes (S2B Fig). Similarly, we detected putative remnant muscles stripes in J4 females using TEM (Fig 3D). These data suggest that muscles do not completely degenerate by J4.

J4 males renew body wall muscle. While lacking the typical organization of mobile J2s or adult males, J4 male phalloidin-stained muscle is substantially larger compared with J3s and J4 females (Fig 2). Examination of J4 male muscles by TEM revealed identifiable sarcomeres, albeit less organized and smaller than in mobile J2s (Fig 3C and Table 1). Like J3 and J4 females, the muscle tissue of J4 males was located more internally than in mobile J2s or adult males, indicating that muscles had not reattached to the body wall (Table 1). When males reach adulthood, the muscles are positioned directly adjacent to the body wall (Fig 3E). The relative size of body wall muscles in adult males is larger compared to J3s or J4 females and comprises well-defined sarcomeres (Figs 2 and 3E and Table 1). Together, our light and electron microscopy data illustrate that *H*. *glycines* undergoes cell-specific muscle atrophy and sex-specific muscle regrowth during development.

### *H*. *glycines* motor neurons degenerate during development

**Fig 4:**
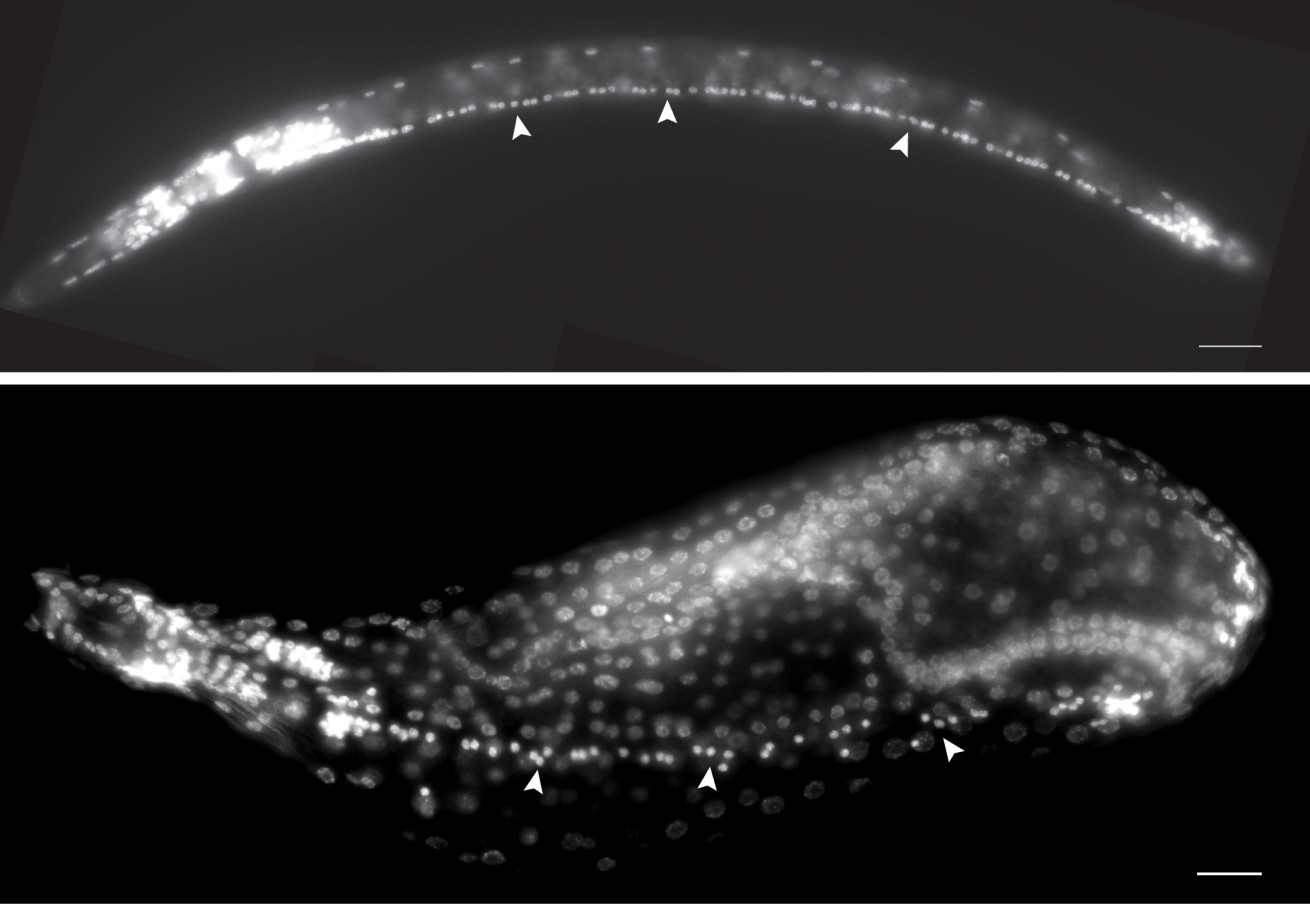
Motor neurons degenerate in the ventral nerve cord during *H*. *glycines* development. Lateral and sub-ventral micrographs of DAPI-stained mobile J2 (top) and sedentary J4 female (bottom), respectively. VNC neuronal nuclei (arrowheads) are highly condensed fluorescent puncta. VNC nuclei in immobile J4 females deviate from the linear pattern seen in mobile J2s.

**Fig 5:**
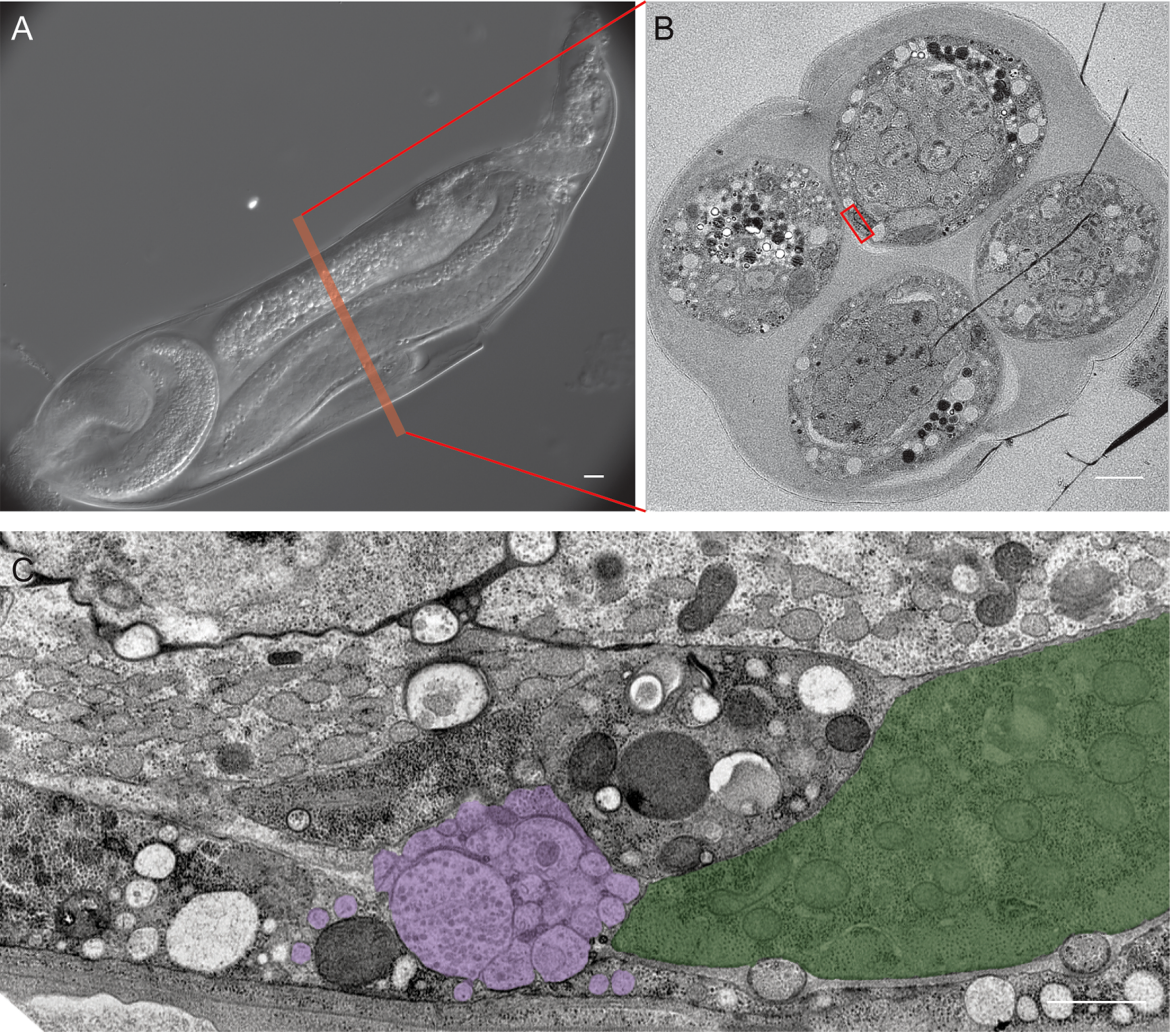
J4 male *H*. *glycines* undergo extensive remodeling. (A) DIC micrograph of J4 male remodeling from sausage-shaped J3 to vermiform adult. At this stage the male is wrapped within the J3 cuticle. (B) Transverse TEM micrograph of J4 male at approximate location of box in (A). Note that the TEM micrographs are not of the same animal as in (A). TEM demonstrate the various parts of the male during J4 are wrapped within a thick extracellular matrix. (C) High magnification of boxed region in (B) showing the VNC (purple) with synaptic vesicles and adjacent body wall muscle (green). The epidermis (tan) at this time point is much thicker than during the mobile J2 or the adult male stage (see Table 1). Scale bar, 1 μm.

In *C*. *elegans*, contraction and relaxation of most body wall muscles are regulated by motor neurons within the ventral nerve cord (VNC) [8,27]. We, therefore, examined the VNC during *H*. *glycines* development. We previously found that mobile J2 *H*. *glycines* contain 65 VNC neurons [12,28]. Here, using DAPI staining we found a gradual reduction of VNC neurons during development from the mobile J2 to sedentary J3 and J4 females (Fig 4 and Table 2). In addition, the overall pattern of the VNC in sedentary stages deviates from the linear pattern seen during mobile stage. Some nuclei of the VNC in sedentary stages are located several microns away from the ventral midline (Fig 4). Examination of the VNC by TEM also suggested a loss of fasciculation and separation from the nearest muscle during J3, while J4 males had a properly fasciculated VNC with synaptic vesicles in several processes (Fig 5). Strikingly, we found 70 neurons in the adult male VNC and a reorganization of the cord into a linear arrangement (Table 2). It appears that, similar to body wall muscles, the motor neurons in the VNC of both sexes of *H*. *glycines* degenerate during sedentary stages of development. However, males undergo neuronal remodeling that includes the addition of neurons. This addition of neurons results in more VNC neurons in adult males than in mobile J2s. Similar to *C*. *elegans*, the males of *H*. *glycines* may include sex-specific motor neurons in the VNC [29,30].

**Table 2:** Developmental changes to the ventral nerve cord in sedentary and mobile nematodes.

Nematode species	Stages	Average number of neurons in VNC^1^	Range	Sample size
*H*. *glycines*	J2^2^	65	59–70	20
	J2 Post-infection	63	56–68	15
	J3 male	53	48–58	15
	J3 female	45	40–54	11
	J4 female	40	32–52	20
	Adult male	70	62–81	14
*M*. *incognita*	J2	59	57–65	10
	J2 Post-infection	50	44–56	9
	Molting to J3	41	35–47	9
	J4/adult female	43	37–47	10
*P*. *penetrans*	J2	58	53–60	10
	J3	58	53–61	8
	J4 female	59	54–62	10
	Adult female	59	54–62	10
	Adult male	63	59–68	8

^1^ Neurons were counted between the retrovesicular ganglion and the preanal ganglion.

^2^ From previously published data [12,28].

### Changes to the GABAergic nervous system during development

**Fig 6:**
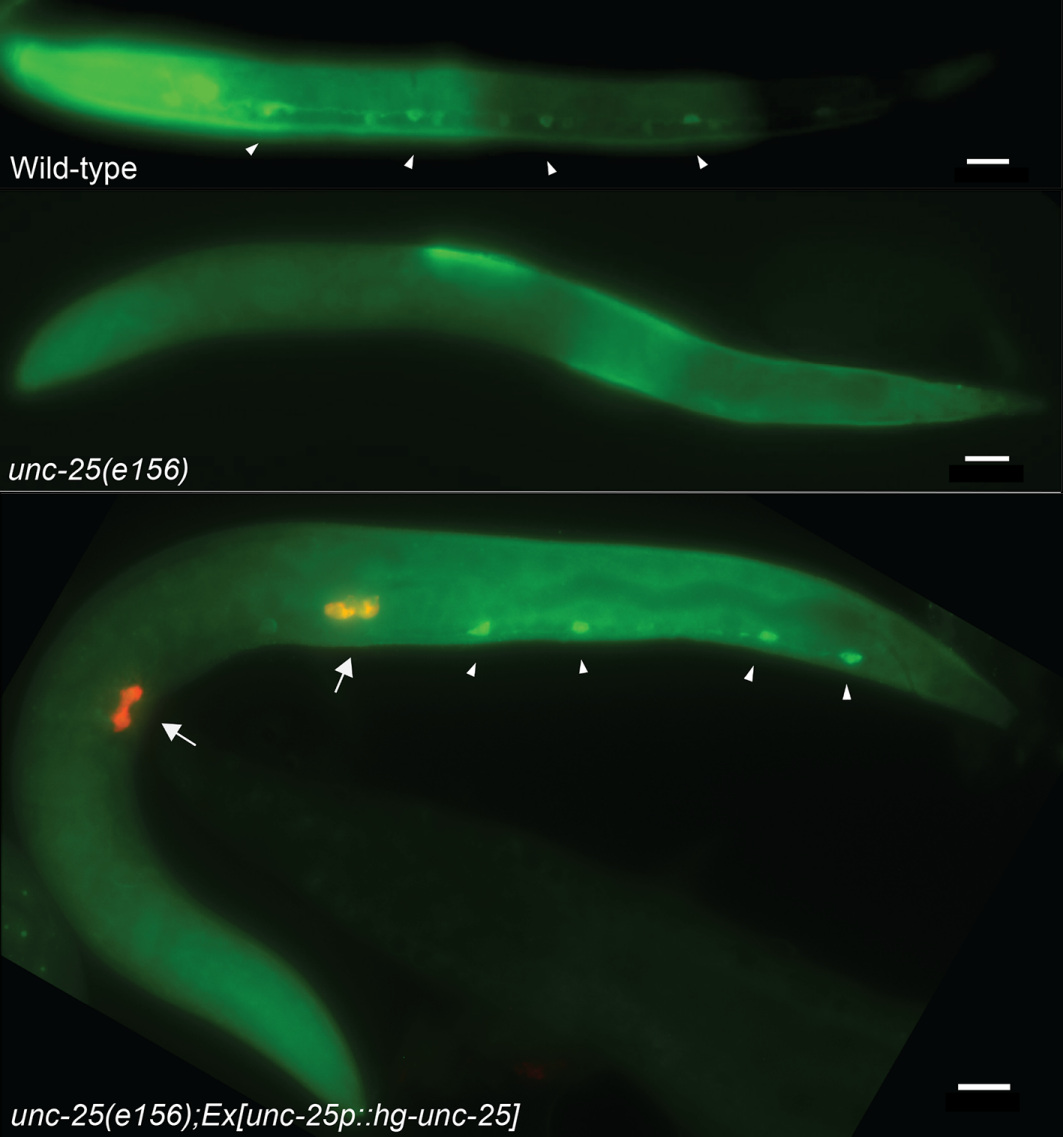
hg-UNC-25 synthesizes GABA in *H*. *glycines*. The *hg-unc-25* cDNA from *H*. *glycines* partially rescues the *C*. *elegans unc-25(e156)* mutant. In wild-type *C*. *elegans*, GABAergic neurons are detected in the head and ventral nerve cord (arrowheads) using anti-GABA staining. In the *C*. *elegans unc-25(e156)* mutant, GABA is not produced and GABAergic neurons cannot be detected using anti-GABA staining. A plasmid construct containing *hg-unc-25* cDNA driven by the *C*. *elegans unc-25* promotor partially rescues the *C*. *elegans unc-25(e156)* mutant as seen by GABA-immunoreactivity (arrowheads). Arrows indicate the expression of *coel*::*RPF* used as a co-injection marker. Scale bars, 10 μm.

To further examine the role of motor neuron degeneration in the sedentary behavior of *H*. *glycines*, we characterized the GABAergic nervous system. In *C*. *elegans*, GABA is produced by approximately one-fourth of the *C*. *elegans* VNC neurons. Release of GABA in the VNC leads to relaxation of body wall muscles and acts in opposition to the excitatory neurotransmitter acetylcholine. GABA is synthesized by glutamate acid decarboxylase (GAD), which in *C*. *elegans* is encoded by *unc-25* [14,15,31]. We cloned the putative ortholog of *unc-25*, from *H*. *glycines* (*hg-unc-25*). The predicted amino acid sequence of *hg-*UNC-25 is over 60% identical to *C*. *elegans* UNC-25 (S3 Fig). To determine if *hg-unc-25* is functional, we attempted to rescue the *C*. *elegans unc-25(e156)* mutant with the *H*. *glycines* homolog. *unc-25(e156)* mutants are completely defective for production of GABA [14]. Using immunohistochemistry, we found that *unc-25(e156); hg-unc-25(+)* animals contained 2–4 GABA VNC, whereas *unc-25(e156)* mutants are completely negative for GABA-immunoreactivity (Fig 6). While our data suggest that hg-UNC-25 functions to produce GABA, wild type *C*. *elegans* contain 19 GABA-immunoreactive neurons. The lack of complete rescue may be due to mosaic expression of the rescuing construct or the absence of unidentified *C*. *elegans-*specific exonic regulatory regions in *hg-unc-25*.

**Fig 7:**
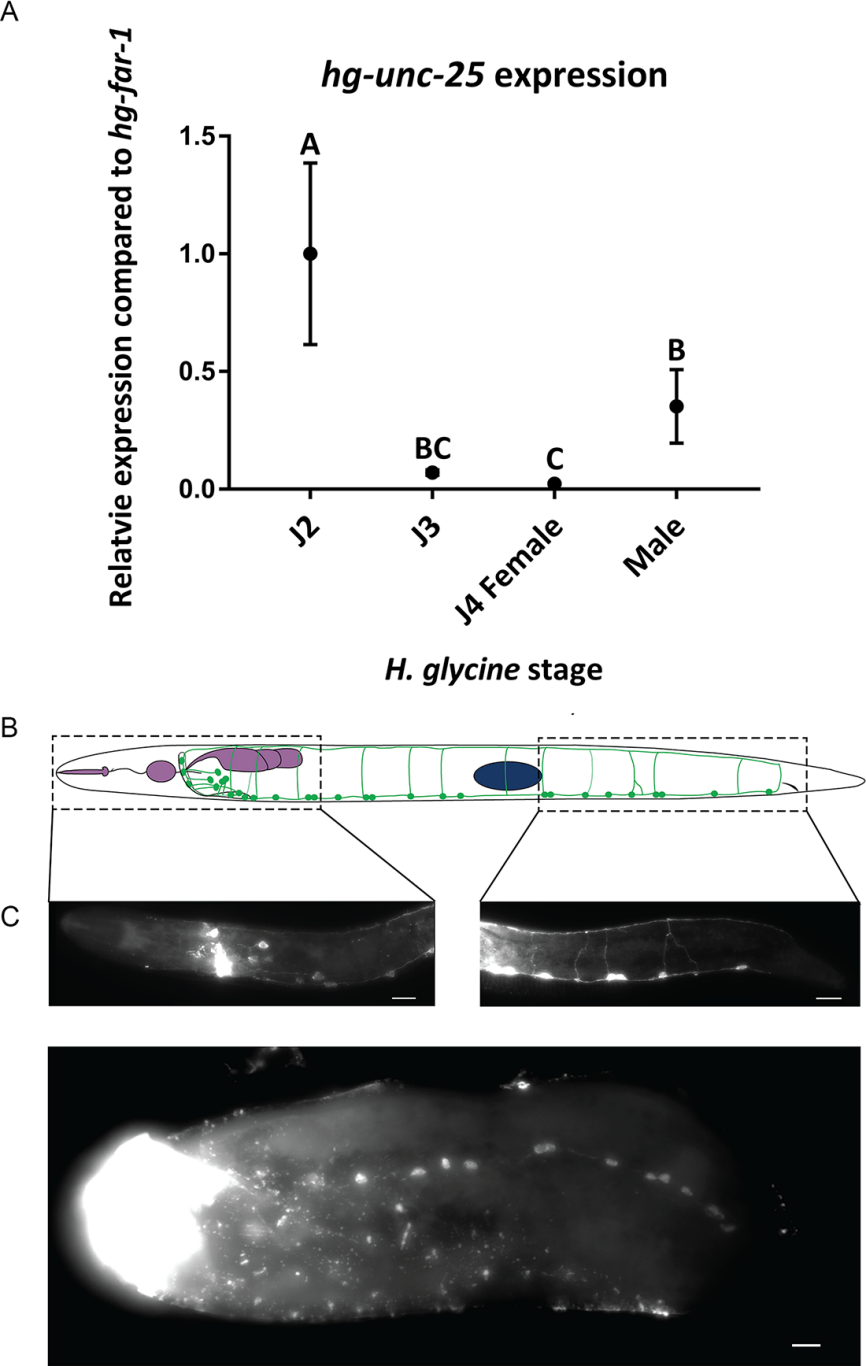
*hg-unc-25* is down regulated and fewer GABA-immunoreactive neurons are detected at the sedentary stages. (A) Relative mean ± standard error expression of *hg-unc-25*, based on RT-qPCR. Data were normalized to J2 with *hg-far-1* used as an internal control. Treatments with different letters are statistically different at α = 0.05 as determined by ANOVA. (B) Lateral left view cartoon (top) and representative fluorescent micrographs of GABA-immunostained J2 *H*. *glycines* sections (bottom). GABA immunostaining was conducted on bisected animals to facilitate penetration. The location of individual neurons was determined by their position relative to landmarks such as the esophagus (purple) and the primordial gonad (blue). GABA-immunoreactive neurons (green) are located surrounding the nerve ring, between the metacorpus and the esophageal glands, and along the VNC. (C) Ventral view fluorescent micrograph of anti-GABA stained *H*. *glycines* J4 female. Scale bars, 10 μm.

We hypothesized that GABAergic VNC neurons were among those that degenerated during development. Therefore, we expected a reduction of *hg-unc-25* expression in sedentary stages compared to the mobile J2s. Using RT-qPCR, we found that expression of *hg-unc-25* is significantly reduced in J3s and J4 females compared to J2s (Fig 7A). These data suggest a reduction in GAD synthesis and possibly a concomitant reduction in GABA production. While *hg-unc-25* expression levels in mobile adult males did not return to levels in mobile J2s, expression was significantly higher in the adult males compared to J4 females.

To examine GABA more directly, we utilized immunohistochemistry to characterize the GABAergic nervous system of *H*. *glycines*. We found that the pattern and morphology of GABA-immunoreactive neurons in J2 *H*. *glycines* were similar, but not identical to *C*. *elegans* [31]. We detected several GABA-immunoreactive cells in the head of *H*. *glycines* surrounding and posterior of the nerve ring that we identified as likely homologs of the RME motor neurons and the polymodal neuron AVL, respectively (Fig 7B). In addition, we identified a pair of GABA-immunoreactive neurons in the head with no known GABAergic positional homolog in *C*. *elegans*. We found 21 GABA-immunoreactive cells in the VNC of J2s (Fig 7B). Similar to *C*. *elegans*, the commissures derived from these GABA-immunoreactive neurons in *H*. *glycines* run to the dorsal side [8]. However, unlike the VNC commissures of *C*. *elegans* and *Ascaris suum*, which usually run along the right side [8,32], we found that the majority of GABA-immunoreactive commissures in *H*. *glycines* travel dorsally along the left side of the animal suggesting a reversal in neuronal handedness. While we found fewer GABA-immunoreactive neurons in the VNC of J4 females ($\bar{x}=12$, n = 12) compared with J2s (Fig 7C), the results were inconsistent. Many individuals showed no antibody staining. Furthermore, the established antibody staining method for plant-parasitic nematodes [33] requires bisecting of the nematode for increased penetration, which, combined with the large size of the J4 female, makes identification of individual neurons and comparison with the J2 stage impossible. Similarly, and for unknown reasons, we were unsuccessful in our attempts to observe GABA-immunoreactivity in adult males. A final caveat to our immunohistochemistry studies is that we cannot differentiate true GABAergic neurons from those that potentially take up GABA. However, combined with our RT-qPCR data, our results suggest that GABAergic neurons are among those that degenerate during sedentary stages.

### Developmentally-associated muscle atrophy and neurodegeneration evolved in phylogenetically separate parasitic nematodes

**Fig 8:**
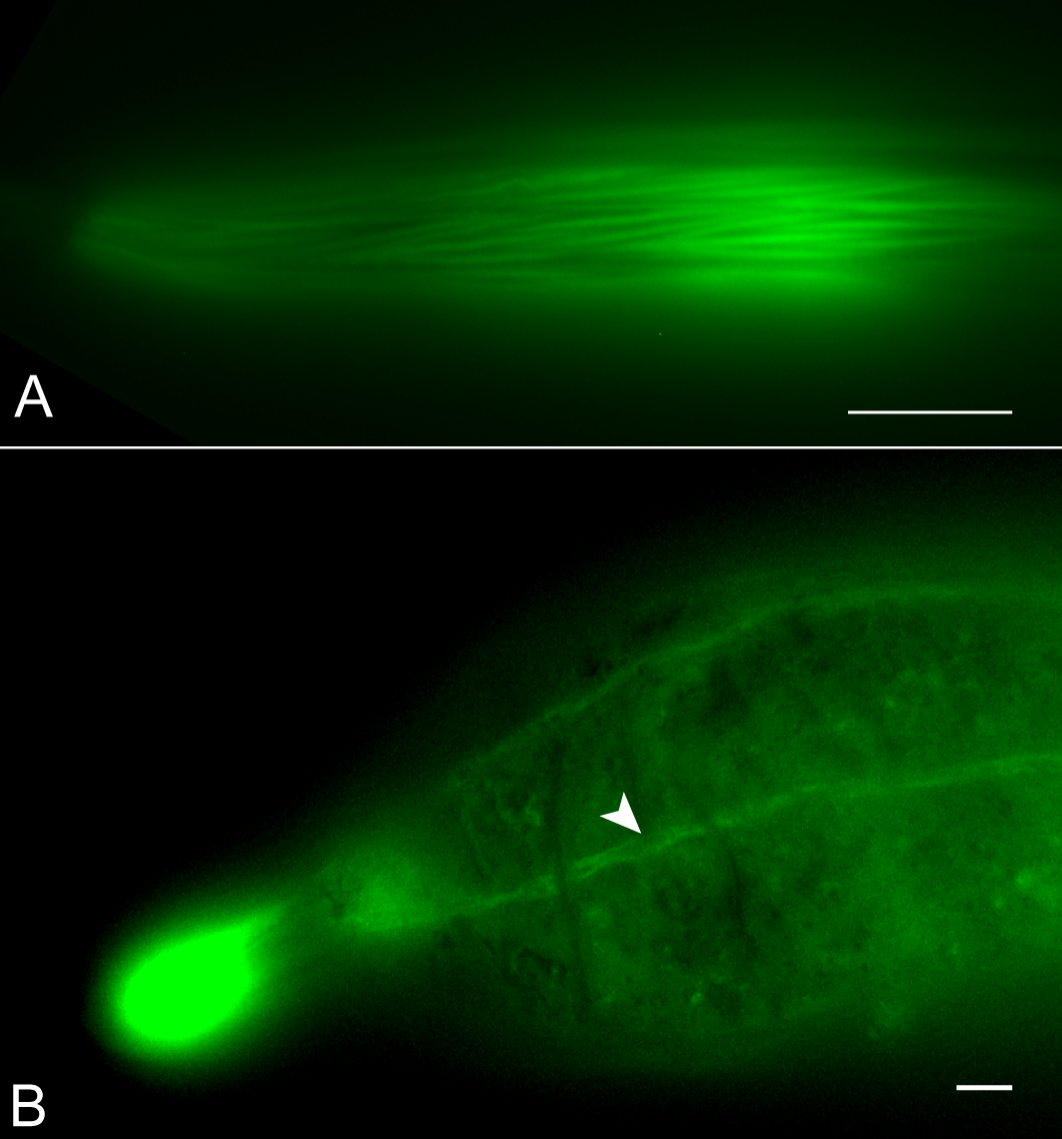
The independently evolved sedentary nematode *M*. *incognita* undergoes muscle atrophy during development. Fluorescent micrographs of phalloidin-stained *Meloidogyne incognita* mobile J2s (A) and sedentary post-infection stage (B). Similar to *H*. *glycines*, *M*. *incognita* undergoes cell-specific muscle atrophy with only remnant body wall muscle stripes (arrowheads) apparent in sedentary stages. Scale bar, 10 μm.

Among Tylenchomorpha nematodes, sedentary behavior is found in multiple genera. The root-knot nematodes (*Meloidogyne* spp.) also become immobile soon after infecting the plant host [6]. However, *Meloidogyne* spp. are phylogenetically diverged from *Heterodera glycines* (S4 Fig). Sister lineages to both *Meloidogyne* spp. and *Heterodera* spp. are mobile during all post-embryonic stages [16,17]. We examined mobile and immobile developmental stages of *Meloidogyne incognita* with light microscopy to determine if changes in neuromuscular structure are correlated with sedentary behavior in phylogenetically diverged species. Similar to *H*. *glycines*, mobile J2 *M*. *incognita* have well developed body wall muscles that atrophy following infection and the onset of sedentary stages (Fig 8). Additionally, we found fewer VNC neurons in sedentary stages of *M*. *incognita* than in the fully mobile J2 (Table 2). Finally, as a control group, we examined the ventral cord of *Pratylenchus penetrans* at different developmental stages. *P*. *penetrans* is classified in the same phylogentic clade as *H*. *glycines* and *M*. *incognita* [16,17], but is mobile at all stages (S4 Fig). We found that the number of ventral cord neurons in *P*. *penetrans* remained stable throughout development. Our current results with *P*. *penetrans*, combined with our previous results with the fungal-feeding Tylenchomorpha nematode *Apehelenchus avenae*, which shows no degeneration of VNC neurons in late development [12], suggest that VNC degeneration in *H*. *glycines* and *M*. *incognita* is specifically correlated with sedentary behavior.

## Discussion

We demonstrate that *H*. *glycines* undergoes a progressive atrophy of neuromuscular tissue that likely leads to immobility. Elsea (1951) suggested that the absence of body wall muscle in *M*. *hapla* adult females was “a result of the sedentary mode of life”; however, this conclusion was based on light microscopy of mobile J2s and adult females. While we cannot entirely rule out that the loss of muscle mass in *H*. *glycines* is caused by a lack of movement, our data suggests this is highly unlikely. We demonstrate that muscle atrophy occurs in conjunction with loss of mobility. Furthermore, cyst nematodes, such as *H*. *glycines*, are able to survive as fully developed, but inactive J2s, for years in a pre-hatch diapause state [34]. Upon activation by a hatching stimulant, these animals readily resume movement suggesting that inactivity alone does not result in muscle atrophy. We suggest that the combination of muscle atrophy and detachment of muscle from the cuticle combined with changes to neuron structure and gene expression cause immobility in feeding cyst nematodes.

The GABAergic system of *C*. *elegans* is essential for proper movement [14]. Our rescue of the *C*. *elegans unc-25* mutant with *hg-unc-25* demonstrates its functional conservation and corresponds with recent data demonstrating the conservation of regulatory regions of *unc-25* among diverse nematode species [35]. Despite the apparent conservation of *unc-25* between *C*. *elegans* and *H*. *glycines*, we found differences in the number and morphology of GABA-immunoreactive cells between these species. Interestingly, we also found that the handedness of most *H*. *glycines* GABA-immunoreactive neurons in the VNC differs from *A*. *suum* and *C*. *elegans*. Our results suggest that nematodes may be an appropriate animal phylum for exploring the evolution of neuronal handedness.

The mechanisms regulating neuromuscular remodeling in *H*. *glycines* are unknown. Previous RT-PCR data of several genes encoding muscle-related proteins showed reduced expression in adult females compared with mobile J2s [5]. A reexamination of the expression of these genes during remodeling may provide insight into the molecular basis of neuromuscular atrophy in *H*. *glycines*. Our data suggest that neuromuscular changes in *H*. *glycines* are cell specific. The absence of atrophy in head and esophageal muscles in *H*. *glycines* allows for continued feeding and head movements during the sedentary stages. In *C*. *elegans*, disruption of the BAG2 chaperone homolog UNC-23 leads to the opposite phenotype wherein the nematode undergoes a progressive degeneration of head muscles [19]. It is possible that cell autonomous changes in expression of *H*. *glycines* muscle-related genes result in the different developmental fates of head and body wall muscles. Alternatively, the muscle cell-specific atrophy seen in *H*. *glycines* could be due to selective neuronal degeneration. The head and neck muscles of *C*. *elegans* are differently innervated than the rest of the body wall muscles [11]. Whereas most *C*. *elegans* body wall muscles are innervated by the VNC, the head muscles receive innervation directly from the circumpharyngeal nerve ring. It will be interesting to determine via TEM whether a similar wiring pattern occurs in *H*. *glycines* and if muscle atrophy is due to denervation as seen in some mammals [36].

The ability to establish a specialized feeding site in their host is a significant adaptation among plant-parasitic nematodes and is often correlated with sedentary behavior. The atrophy of neuromuscular tissue is likely due to a relaxation of selection pressure for the maintenance of mobility. In addition, the degeneration of neuromuscular tissue could allow energy derived from the catabolism of muscle protein to be shunted toward reproductive development. The presence of muscle atrophy in both *H*. *glycines* and *M*. *incognita* strongly suggests the independent evolution of this behavior. However, it is important to note that within the Tylenchida, which includes both plant and insect parasitic nematodes, sedentary behavior is found in several phylogenetically separate genera [16]. It is, therefore, possible that sedentary behavior evolved first in a basal lineage within the Tylenchida and was repeatedly lost during evolution. Careful comparative analysis of these genera will be required to fully resolve these competing hypotheses.

## Materials and methods

### Nematode culture

*H*. *glycines* was originally isolated from a soybean field in Illinois, USA and cultured in the greenhouse on soybean cultivar ‘Macon’. To collect synchronized developmental stages of *H*. *glycines*, seeds of the soybean variety ‘Macon’, were germinated in a moist paper towel for three days. Soybean seedlings were then placed in pluronic gel F-127 with hatched J2 *H*. *glycines* for 24 hours [37]. Alternatively, soybean seedlings were planted into pots and inoculated with freshly hatched *H*. *glycines* second-stage juveniles (J2s). After 24 hours, roots were washed with water to remove nematodes that had not infected. *H*. *glycines*-infested roots were then planted into a pasteurized sandy loam soil and kept under a 12-hour light cycle until extraction. Infested roots were macerated with a hand blender to obtain *H*. *glycines* at specific time points. The roots were processed 6–7 days after inoculation to collect J3s and 9–10 days after inoculation to collect J4 females and males [38]. Developmental stages and sex were determined by overall body size and gonad morphology [23]. *Meloidogyne incgonita* was a gift from Dr. Jason Bond and maintained on ‘Rutgers’ tomato in the greenhouse. Seeds of tomato variety ‘Rutgers’ were planted into a sandy loam soil for two weeks and then inoculated with freshly hatched *M*. *incognita* J2s. After four weeks, post-infective stages of *M*. *incognita* were extracted from the tomato plants and the developmental stages were identified based on morphology [39]. *P*. *penetrans* was a gift from Dr. Terry Niblack and was cultured on corn root explants on Murashige and Skoog (MS) media [40]. Specific developmental stages of *P*. *penetrans* were isolated by synchronizing populations from eggs. *P*. *penetrans* eggs were extracted as previously described [41] Freshly hatched J2s were collected and placed on corn root explants on MS media [40]. Developmental stages were determined by the presence of molting, gonad morphology and overall body size. *C*. *elegans* strains N2 and CB156 *unc*-*25(e156)* III were cultured on NGM agar with *E*. *coli* OP50 as previously described [42].

### Phalloidin staining

Nematodes were fixed in 4% paraformaldehyde (Electron Microscopy Science) overnight at 4°C and washed three times with water. Nematodes were placed on a 4% agar pad with phalloidin (10 unit/ml; Thermo Fisher Scientific) and DAPI (0.2–0.5 μg/ml; Thermo Fisher Scientific) or Hoechst (0.2 mM; Thermo Fisher Scientific) to stain nuclei. An Andor system micropoint laser attached to a Zeiss Axioimager with 63x objective was used to create openings in the cuticle at regular intervals along the length of the nematode to increase the penetration of dyes. Following opening of the cuticle, nematodes were incubated in the stain overnight. Successful penetration of phalloidin in sedentary nematodes was confirmed by observing fluorescence in esophageal muscle. At least ten animals were examined for each developmental time point. All light microscopy images were captured with Zen software on a Zeiss M2 AxioImager with DIC and fluorescence optics. Images were examined in FIJI and multiple images were combined using the ImageJ stitching plugin.

### DAPI staining

Nematodes were fixed in Carnoy’s fixative (60% ethanol, 30% acetic acid, 10% chloroform) overnight in a 1.5 ml centrifuge tube and allowed to settle in the bottom of the tube [43,44]. The supernatant was removed and transferred to 75% ethanol before staining with 0.2–0.5 μg/ml of DAPI overnight in the dark at room temperature. The VNC nuclei were identified based on their size and morphology [12,45]. At least eight animals were examined for each species at each developmental time point.

### Electron microscopy

Synchronized developmental stages of *H*. *glycines* were collected from soybean roots and stored overnight at 4°C. High pressure freezing and freeze substitution were modified from previous methods used for *C*. *elegans* [46,47]. Metal specimen carriers were coated with 1-hexadecene and a layer of *E*. *coli* strain OP50. Nematodes were loaded into carriers with 20% bovine serum albumin and frozen in an HPM 010 high pressure freezer. Freeze substitution was performed in 2% OsO_4_ (Electron Microscopy Sciences) and 0.1% uranyl acetate (Polysciences) in 2% H_2_O and 98% acetone in an FS-8500 freeze substitution system. Samples were kept at -90°C for 110 hours before being warmed to -20°C over five hours. Samples were then kept at -20°C for 16 hours before being warmed to 0°C over five hours. Samples were washed four times in pre-chilled 100% acetone at 0°C. The last wash was one hour. Samples were then transferred to room temperature and washed two times in 100% acetone. Samples were infiltrated with 1:1 Polybed812 (Polysciences) resin:acetone for 24 hours, 2:1 resin:acetone for 36 hours, 100% resin for 24 hours, and then changed to fresh resin for three days. All infiltration steps were conducted on an orbital shaker at room temperature. Samples were then submerged into embedding molds with resin and hardener and baked at 60°C for two days. 70 nm sections were collected using a PowerTome PC ultramicrotome with a diamond knife and collected onto formvar-coated copper slot grids. Sections were imaged with a Phillips CM200 TEM. Quantification of sarcomere size, epidermal thickness, and body area were calculated using FIJI measurement tools. Pseudocolor overlays were created with Adobe Photoshop using the Wormatlas tissue color code scheme (http://www.wormatlas.org/colorcode.htm). Multiple sections were examined from at least two animals at each time point.

### Antibody staining

*C*. *elegans* and *H*. *glycines* were fixed in 4% paraformaldehyde and 2.5% glutaraldehyde (Electron Microscopy Science) for 15 minutes at 4°C. Fixed nematodes were washed three times with PBST (8 mM Na_2_HPO_4_, 150 mM NaCl, 2 mM KH_2_PO_4_, 3 mM KCl, 0.05% Triton X-100, pH 7.4) [31]. To increase the permeability of fixed *C*. *elegans*, nematodes were washed three times after fixation with PBST and shaken in 5% β-mercaptoethanol/PBST (Sigma-Aldrich) at 37°C overnight [31]. *C*. *elegans* were then washed three times with 1 mM CaCl_2_/1% Triton X-100/0.1 M Tris-HCl (pH 7.5). 200 U/ml collagenase type IV was added to *C*. *elegans* and shaken vigorously for 15–30 minutes at 37°C, and washed three times with PBST. To increase the permeability of fixed *H*. *glycines*, specimens were cut into small segments [33]. *H*. *glycines* sections were incubated in 100 mM Tris/1mM CaCl_2_ and proteinase K (2 mg/ml) for 20–30 minutes at room temperature to facilitate antigen retrieval. Nematode segments were incubated in prechilled methanol on ice for one minute followed by one minute on prechilled acetone on ice. Segments were washed three times with PBST. After permeabilization, both nematode species were incubated in 1% BSA (Sigma-Aldrich) dissolved in PBST for at least one hour followed by incubation in a 1:100 anti-GABA (rabbit; Sigma-Aldrich) primary antibody at 4°C overnight. Nematodes were washed three times with PBST and incubated in 0.1%BSA/PBST with a 1:100 secondary anti-rabbit IgG-FITC (goat; Sigma-Aldrich) antibody at a 1:100 ratio overnight before examination. Antibody staining was observed on over 40 J2 *H*. *glycines* and 10 sedentary *H*. *glycines*.

### *hg-unc-25* rescue construct *unc-25p*::*hg-unc-25* and microinjection

Fresh *H*. *glycines* J2s were homogenized in liquid nitrogen to extract RNA using the Qiagen RNeasy Blood Kit (Qiagen) following the manufacturer’s instruction. Genomic DNA contamination was removed from the RNA using the Turbo DNA-free kit (Thermo Fisher Scientific). cDNA was synthesized using the Thermoscript RT-PCR system (Invitrogen). *hg-unc-25* was amplified from *H*. *glycines* cDNA using primers 5’-ATGAAATTAAAGGAGCATAAAGAATC-3’ and 5’-TCACAAATCTTCTCCCAGTGTG-3’. The plasmid pPD157.60 (gift from Andrew Fire via Addgene) was used as the backbone for the construct. Forward: 5’-CATTTTTTCTACCGGTACCAATACG-3’ and reverse: 5’- ACATTTTTTTTTCTCTTTCCGTCTC-3’ primers were used to amplify the backbone including a 1.9 Kb *unc-25* promoter region from pPD157.60 (Addgene). The backbone and *hg-unc-25* cDNA were ligated using Gibson Assembly (ThermoFisher Scientific) following the manufacturer's instruction. Three 51-bp artificial introns were added into the *hg-unc-25* cDNA to improve expression (Andrew Fire Vector Kit) using Gibson Assembly. The rescue construct *unc-25p*::*hg-unc-25* (40 ng/ul) was co-injected with the coelomocyte marker plasmid *coel*::*RFP* (80 ng/ul) to rescue the *C*. *elegans unc-25(e156)* mutant using standard microinjection techniques [48]. Five independent stable lines were examined for rescue of GABA immunoreactivity. At least 30 nematodes from both the rescue lines and controls were examined.

### RT-qPCR on the expression of *hg-unc-25* in *H*. *glycines*

Synchronized developmental stages of *H*. *glycines* were homogenized in liquid nitrogen. RNA was extracted and genomic DNA contamination was removed from the RNA. RT-qPCR was performed using the Power SYBR Green RNA-to-Ct 1 step kit on an ABI PRISM 7000 Sequence Detection System. Expression of *hg-unc-25* was examined using the primers qhgunc25 forward primer 5’-TCCAAAGGGATGGAAGGTTATC-3’ and reverse primer 5’-GCCTTCAGTGCGTTTGATTT-3’. *far-1*, a surface associated retinol and fatty-acid protein encoding gene was compared for the relative expression of *hg-unc-25* [49,50]. Each treatment group comprised three independent replicates and the experiment was repeated twice. Graphpad Prism 6 was used for statistical analysis. For the relative expression of *hg-unc-25*, the ΔCt between *hg-unc-25* and *hg-far-1* at each stage was calculated, and a one-way ANOVA was performed to calculate the mean separation [51]. Normalized data are shown.

## Supporting information

S1 Fig Cartoon of J2 *H*. *glycines* with the cuticle and epidermis removed from the subventral quadrant to show the longitudinal organization of body wall muscles. (TIF)

S2 Fig Fluorescent micrograph of (A) phalloidin-stained J3 *H*. *glycines* head region demonstrating that head (arrow) and esophageal (arrowhead) muscles do not degenerate. (B) Phalloidin (green) and DAPI (blue) staining of J4 body wall muscle actin and nuclei. Scale bars, 10 μm.
(TIF)

S3 Fig The amino acid sequence alignment between *C*. *elegans* UNC-25 (isoform a) and *hg-*UNC-25 was performed using multiple sequence alignment in Clustal Omega. In *C*. *elegans*, *unc-25* encodes the sole enzyme glutamate acid decarboxylase for GABA synthesis. *hg-*UNC-25 is 69% identical to UNC-25.
(TIF)

S4 Fig Phylogenetic relationship of nematodes discussed in text based on SSU rDNA sequence [16,17]. (PDF)
