# Supplementary figures and images for: Immobility in the sedentary plant-parasitic nematode H. glycines is associated with remodeling of neuromuscular tissue
Source: PLoS Pathog. 2018 Aug 16;14(8):e1007198. doi: 10.1371/journal.ppat.1007198 (PMC6095618; doi:10.1371/journal.ppat.1007198)

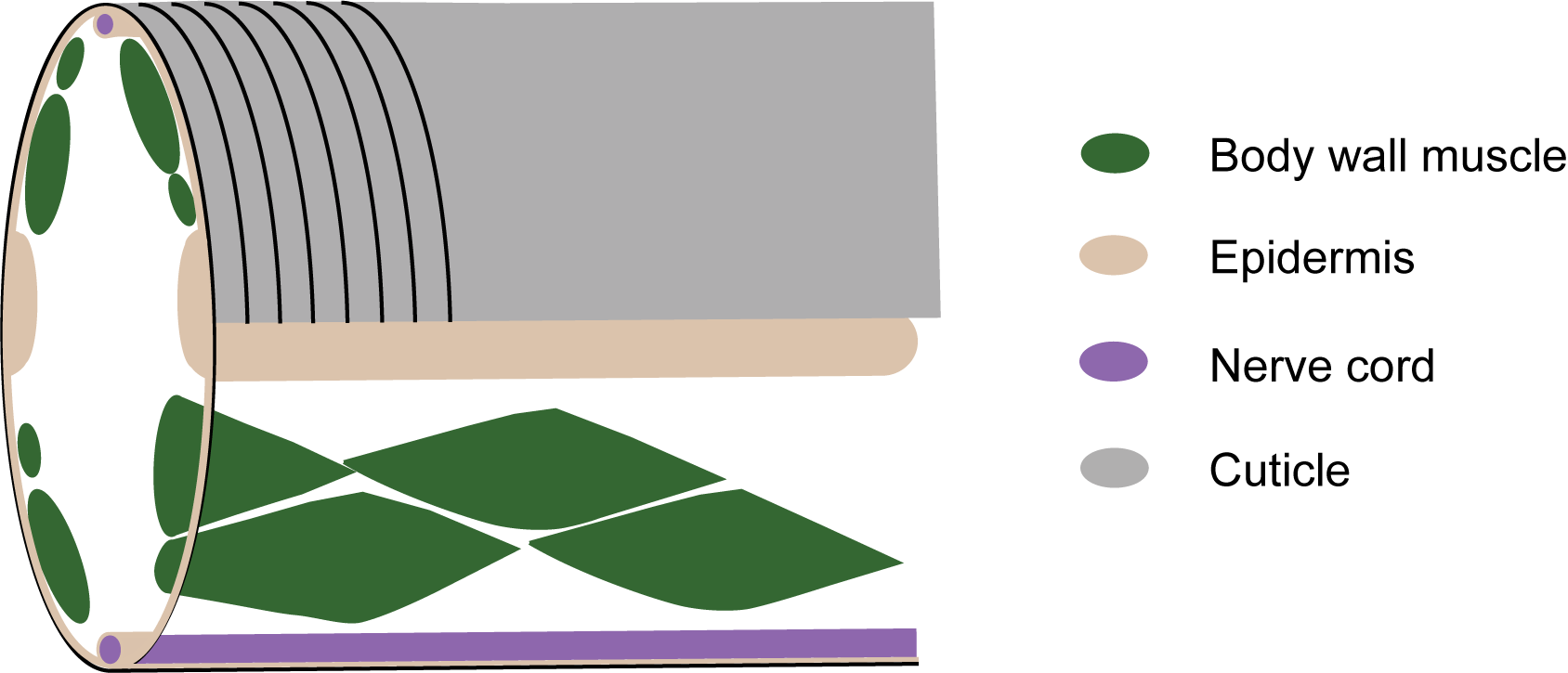

Supplement: S1 Fig — (TIF) [file ppat.1007198.s001.tif]

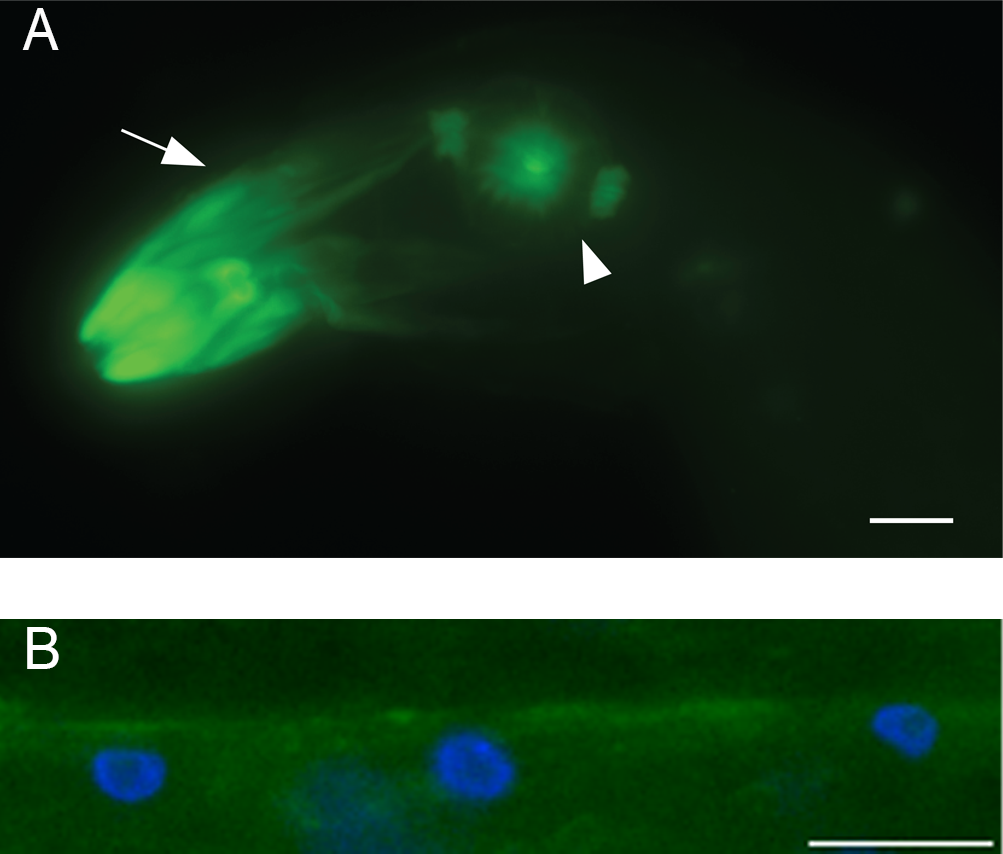

Supplement: S2 Fig — Fluorescent micrograph of (A) phalloidin-stained J3 H. glycines head region demonstrating that head (arrow) and esophageal (arrowhead) muscles do not degenerate. (B) Phalloidin (green) and DAPI (blue) staining of J4 body wall muscle actin and nuclei. Scale bars, 10 μm. (TIF) [file ppat.1007198.s002.tif]

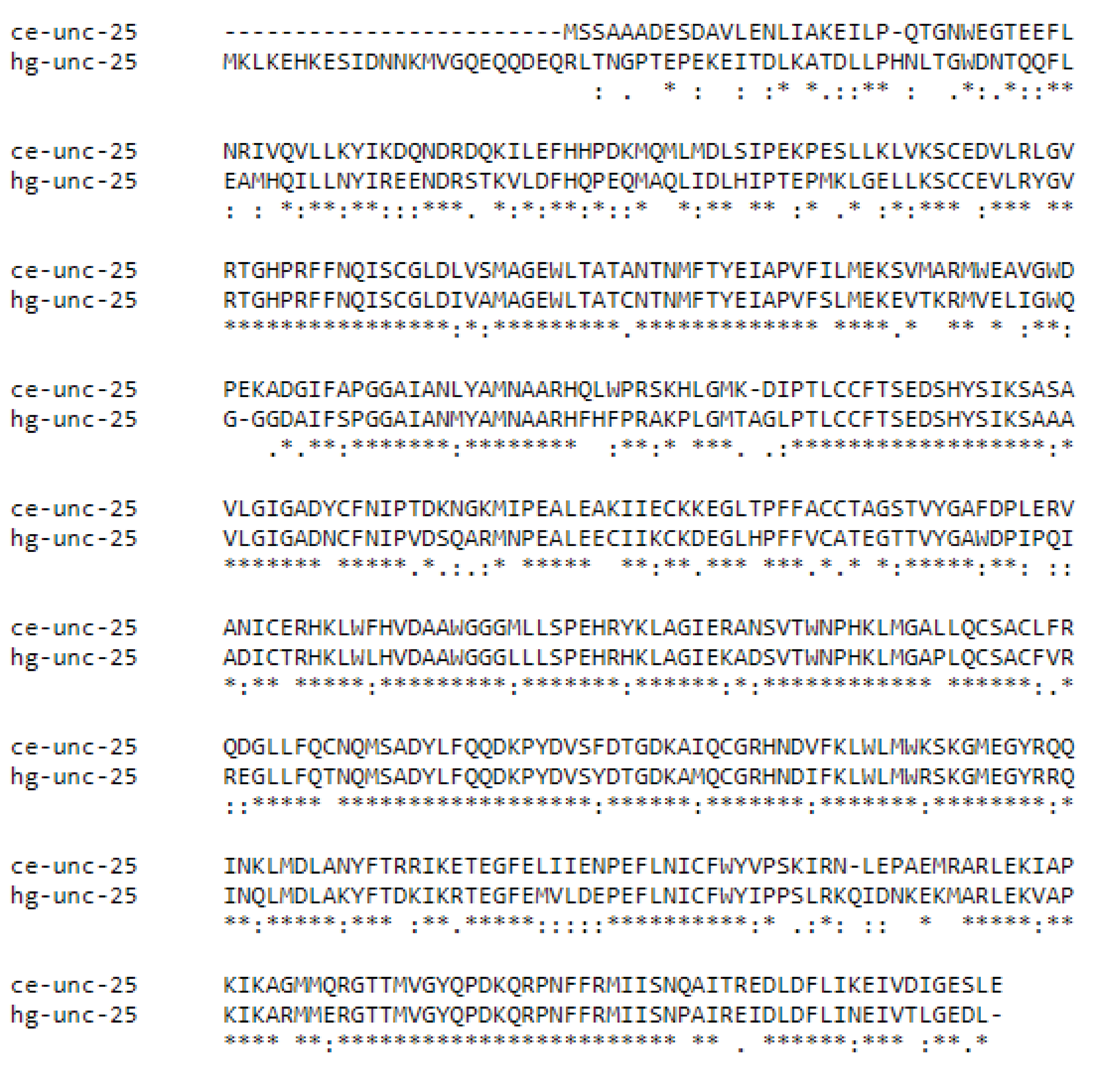

Supplement: S3 Fig — In C. elegans, unc-25 encodes the sole enzyme glutamate acid decarboxylase for GABA synthesis. hg-UNC-25 is 69% identical to UNC-25. (TIF) [file ppat.1007198.s003.tif]

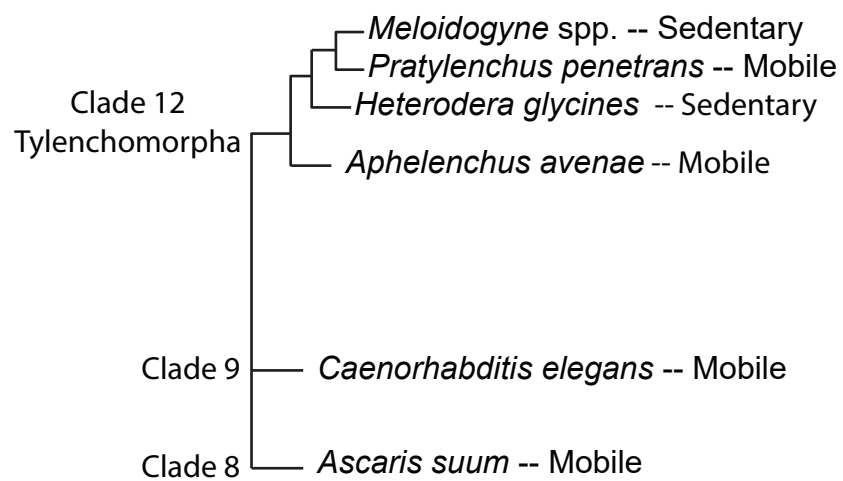

Supplement: S4 Fig — (PDF) [file ppat.1007198.s004.pdf]
